# SAAP‐148 Oligomerizes into a Hexamer Forming a Hydrophobic Inner Core

**DOI:** 10.1002/cbic.202500112

**Published:** 2025-04-21

**Authors:** Aden Hodzic, Djenana Vejzovic, Altea Topciu, Kirill Kuhlmann, Raj Kumar, Maria Andrea Mroginski, Alejandra de Miguel, Pia Hofmann, Klaus Zangger, Markus Weingarth, Robert A. Cordfunke, Jan W. Drijfhout, Peter Nibbering, Michal Belicka, Karl Lohner, Nermina Malanovic

**Affiliations:** ^1^ NanoEntum Ruckerlbergergürtel 10 8010 Graz Austria; ^2^ Institute of Molecular Biosciences University of Graz 8010 Graz Austria; ^3^ NMR Spectroscopy Department of Chemistry Utrecht University Padualaan 8 3584 CH Utrecht The Netherlands; ^4^ Institut für Chemie Technische Universität Berlin Straße des 17. Juni 115 10623 Berlin Germany; ^5^ Institute of Chemistry/Organic and Bioorganic Chemistry University of Graz 8010 Graz Austria; ^6^ Field of Excellence BioHealth University of Graz 8010 Graz Austria; ^7^ BioTechMed Graz 8010 Graz Austria; ^8^ Department of Immunology Leiden University Medical Center 2333 ZA Leiden The Netherlands; ^9^ Laboratory of Infectious Diseases Leiden University Center of Infectious Diseases (LU‐CID) Leiden University Medical Center 2333 ZA Leiden The Netherlands

**Keywords:** antimicrobial peptides, hydrophobic cores, lipid–peptide interactions, peptide aggregates, SAAP‐148

## Abstract

Human cathelicidin LL‐37 derivative, the 24‐mer SAAP‐148, is highly effective *in vitro* in eradicating multidrug‐resistant bacteria without inducing resistance. SAAP‐148 has a high cationic charge (+11) and 46% hydrophobicity, which, once the peptide folds into an alpha helix, forms a wide hydrophobic face. This highly amphipathic nature facilitates on the one hand its insertion into the membrane's fatty acyl chain region and on the other hand it´s interaction with anionic membrane components, which aids in killing bacteria. However, the contributions of the secondary and quaternary structures have not been thoroughly investigated so far. To address this, we applied circular dichroism, NMR spectroscopy, X‐ray scattering, AlphaFold 3 protein folding software, and molecular dynamics simulations. Our results reveal that SAAP‐148 adopts a stable hexameric bundle composed of three parallel dimers, that together form a hydrophobic core of aromatic side chain residues. The hexameric structure is retained at the membrane interface, whereby, MD simulation studies indicated the formation of a fiber‐like structure in the presence of anionic membranes. This certainly seems plausible, as oligomers are stabilized by aromatic residues, and the exposure of positively charged side chains on the surface likely facilitates the transition of the peptide into fibrils on anionic membranes.

## Graphical abstract

The highly cationic and hydrophobic antimicrobial peptide SAP‐148 effectively eradicates multidrug‐resistant bacteria, persistent cells, and biofilms without inducing resistance. Its efficiency is likely due to its ability to disrupt bacterial membranes as an oligomer, forming a stable alpha‐helical hexameric bundle with three parallel dimers that create a hydrophobic core.

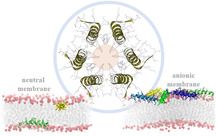

## 1 Introduction

The increasing emergence of antimicrobial resistance and untreatable infections remains one of the biggest threats concerning public health and modern medicine.^[^ ^1^, ^2^, ^3^, ^4^, ^5^, ^6^ ^]^ Most of these infections are caused by multidrug‐resistant (MDR) strains belonging to the so‐called ESKAPE (*Enterococcus fecium*, *Staphylococcus aureus*, *Klebsiella pneumoniae*, *Acinetobacter baumannii*, *Pseudomonas aeruginosa,* and *Enterobacter species*) platform.^[^ ^1^, ^7^, ^8^ ^]^ Such bacteria prefer forming biofilms, typical bacterial aggregates embedded within a self‐established matrix of extracellular substances, adding an additional layer of complexity to the treatment with conventional antibiotics.^[^ ^1^, ^9^, ^10^, ^11^ ^]^ Moreover, mature biofilms tend to form metabolically inactive persisters/dormant bacteria, enabling the recurrence of biofilm‐associated infections.^[^ ^1^, ^11^ ^]^ Altogether, these characteristics of bacterial biofilms largely contribute to the development of the observed antimicrobial resistance. This problem points to an increased need to explore and develop novel effective antimicrobial treatments with diminished induction of bacterial resistance.

Antimicrobial peptides (AMPs) have attracted great interest as potential candidates to combat bacterial infections caused by multidrug resistant pathogens.^[^ ^12^, ^13^, ^14^, ^15^, ^16^, ^17^, ^18^ ^]^ They are naturally produced by all living organisms as important components of their immune system and display a potent and broad‐spectrum activity against numerous classes of bacteria, yeasts, fungi, viruses, and parasites.^[^ ^16^, ^18^, ^19^ ^]^ The added advantage of such molecules is their nonspecific mode of action, namely disruption of fundamental barrier function by permeabilization of the bacterial cytoplasmic membrane, associated with bacterial death, thus a reducing the emergence of bacterial drug resistance.^[^ ^1^, ^20^, ^21^, ^22^ ^]^ However, AMPs’ clinical application is often hindered by several factors including poor stability, cytotoxicity, hemolytic activity, and production costs.^[^ ^16^, ^19^, ^23^ ^]^ Different strategies have been carried out to address this issue and to produce AMP with optimized properties and activity.^[^ ^1^, ^18^, ^19^, ^24^ ^]^ A good example from the toolbox of such compounds is SAAP (Synthetic Antimicrobial and Antibiofilm Peptide)‐148, which is derived from screening a pool of shortened LL‐37‐inspired peptides and substituting Q with cationic R or K.^[^ ^1^ ^]^

SAAP‐148 proved to be highly effective in killing MDR bacteria under physiological conditions in vitro, without inducing resistance.^[^ ^1^ ^]^ In addition, it was able to completely eradicate biofilm‐associated infections with methicillin‐resistant *Staphylococcus aureus* and *Acinetobacter baumannii* in wounded human skin ex vivo and murine skin in vivo, as well as successfully eradicate persisters generated inside different mature biofilm infection models.^[^ ^1^, ^25^ ^]^ Besides its promising therapeutic potential, SAAP‐148 induces some hemolysis of red blood cells at higher concentrations.^[^ ^1^ ^]^ Nevertheless, 10% hemolysis, which represents the cut‐off for measured hemolysis in vitro,^[^ ^26^, ^27^, ^28^ ^]^ is not exceeded at the concentration range, where SAAP‐148 exhibits its antimicrobial activity.^[^ ^1^, ^22^ ^]^ SAAP‐148 displays a safe therapeutic profile in topical treatments on the murine skin in vivo and human skin.^[^ ^1^ ^]^

A general mode of action for SAAP‐148 in both Gram‐positive bacteria and Gram negative bacteria has been earlier proposed in our studies.^[^ ^20^, ^21^, ^22^ ^]^ In Gram‐positive bacteria (e.g., *Enterococcus hirae*), after an initial attachment to the bacterial surface, SAAP‐148 penetrates through the cell wall toward the cytoplasmic membrane.^[^ ^21^ ^]^ Here, its hydrophobic region is embedded into the fatty acyl chains, inducing a so‐called dimple formation.^[^ ^21^, ^22^ ^]^ This is followed by a number of subsequent events, including changes in membrane fluidity, an enhanced membrane permeability, and membrane depolarization, leading to membrane rupture and cell death.^[^ ^21^, ^22^ ^]^

In contrast, in Gram‐negative bacteria (e.g., *Escherichia coli*), SAAP‐148 binds and neutralizes their surface, mainly through electrostatic interactions between the positively charged amino acids and negatively charged bacterial components.^[^ ^20^, ^21^ ^]^ Subsequently, it crosses through the outer membrane and cell wall, until it is again inserted into the fatty acyl chain region of the inner cytoplasmic membrane.^[^ ^21^ ^]^ At this stage, the membrane damage may not necessarily result in membrane rupture, where leakage of only a small amount of ions might actually be sufficient for causing cell death.^[^ ^21^ ^]^

Interestingly, SAAP‐148 exhibits potent activity in killing both Gram‐positive bacteria and Gram‐negative bacteria in comparison to other AMPs (e.g., LL‐37 or OP‐145), which can be ascribed to its superior ability to disrupt bacterial membranes.^[^ ^1^, ^20^, ^21^, ^22^ ^]^

Indeed, helical wheel projections indicate a larger hydrophobic region in the sequence of SAAP‐148.^[^ ^20^, ^22^ ^]^ Additionally, in silico studies of membrane partitioning revealed that SAAP‐148 needs less free energy to transfer form water to the lipid bilayer, supporting the higher membrane destabilization, since SAAP‐148 can penetrate more easily and deeper into the membrane.^[^ ^20^, ^22^ ^]^ This happens due to the presence of specific amino acids in the hydrophobic region of SAAP‐148, namely W5 and W16 which interact with the membrane interface.^[^ ^20^, ^22^ ^]^

Furthermore, presence of the amino acids such as Q18 and Y15 tends to form hydrogen bonding interactions with the phospholipid head groups, increasing the SAAP‐148 efficiency in disrupting bacterial membranes.^[^ ^20^, ^22^ ^]^

However, until now limited information is available regarding the structure of SAAP‐148 in free solution or when present in a membrane interface. Such knowledge is important because it might improve our understanding of the interactions of this peptide with bacterial membranes and subsequently provide valuable insights into its mode of action at molecular level. In addition, such knowledge seems to be crucial for improving the peptide primary structure even further. In this article, we aim to elucidate the formation of the secondary and tertiary structures of SAAP‐48, specifically focusing on its interaction with anionic and zwitterionic model membranes using various technical approaches.

## 2 Results

### 2.1 SAAP‐148 Forms a Hydrophobic Core in Anionic Membrane Vesicles

**Figure 1:**
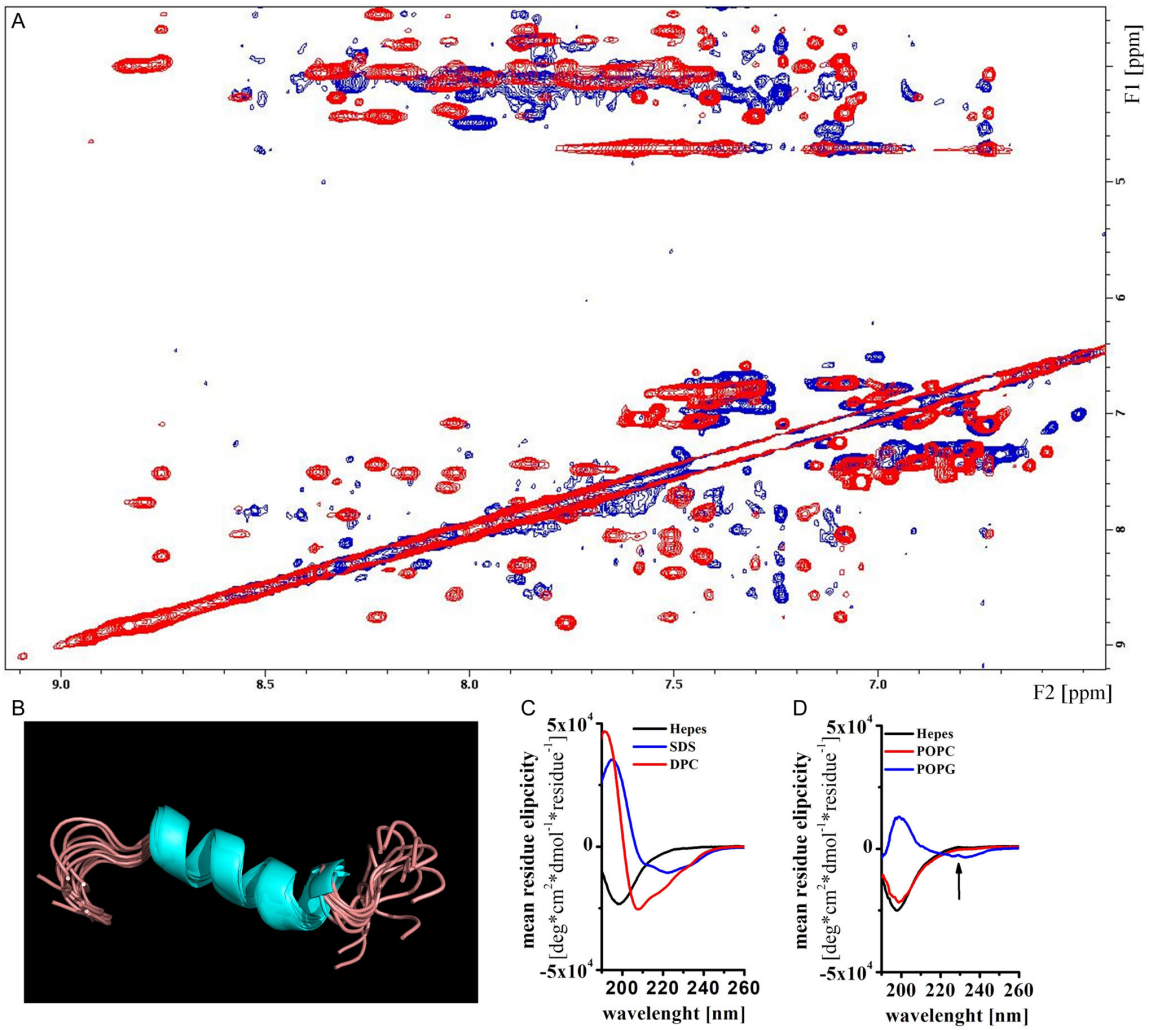
Structural properties of SAAP‐148. A) Overlay of the NH–NH and fingerprint region of the 2D NOESY spectra of SAAP‐148 in DPC (red) and SDS (blue). B) 3D structure of SAAP‐P148 in presence of DPC calculated by CNS and illustrated with the application PyMOL; the image shows the 10 structures with the lowest energy out of 20 accepted calculated structures; the backbone root mean square deviation (RMSD) value is 0.34 Å in the well‐order part of the peptide ranging from residues 4–20. C) CD spectra of 200 μm peptide in 10 mm Hepes (black), in the presence of 5 mm DPC (red) and 5 mm SDS (blue). The data are representative for at least three independent measurements. D) CD spectra of 200 μm peptide in 10 mm Hepes (black), in the presence of 5 mm POPC (red) and 5 mm POPG (blue). The data are representative for at least three independent measurements.

*In silico* studies predicted an alpha helical conformation for SAAP‐148, similarly to that observed for its counterpart LL‐37 and OP‐145.^[^ ^22^ ^]^ To verify the helical structure of SAAP‐148, we performed both NMR spectrometry and circular dichroism (CD) spectroscopy of the peptide in solution. NMR experiments were conducted on the peptide while being present in small lipid micelles, using dodecylphosphocholine (DPC) and anionic sodium dodecyl sulfate (SDS). The measurements revealed the formation of a well‐defined α‐helix from amino acid 6 to amino acid 19 (**Figure** **1**A,B). The overlay of the nuclear overhauser enhancement spectroscopy (NOSY) spectra of SAAP‐148 in DPC and SDS showed correlations between the NH protons (around 8 ppm) and between the Hα (≈4–5 ppm) and NH protons. Numerous strong NH–NH peaks further supported the presence of an α‐helical structure. Additionally, average Hα chemical shifts close to 4 ppm were indicative of α‐helix structure.^[^ ^29^ ^]^ In DPC, SAAP‐148 appeared more structured, as evidenced by a higher number of intense and well‐defined peaks. However, some regions were less well‐defined, suggesting the peptide may form aggregates. Pulsed‐field gradient diffusion NMR measurements of the hydrophobic radii of SAAP‐148 in the presence of micelles revealed an increase in the hydrophobic radius of SDS from ≈25 to 32 Å, indicating aggregation. In contrast, no such changes were observed in DPC, further indicating that aggregation is more likely to occur in the presence of SDS. Furthermore, hydrodynamic radius of SAAP‐148 alone was measured to be ≈15 Å, which further suggests the potential for peptide aggregation in solution assuming an ideal alpha helical peptide to have a radius of aproximately 3‐6 Å, depending on the lenght of the side chains.

For the CD experiments, the peptide's secondary structure was evaluated also in the presence of anionic palmitoyl‐oleyl‐phosphatidylglycerol (POPG) and zwitterionic palmitoyl‐oleyl‐phosphatidylcholine (POPC), which respectively mimic bacterial and eukaryotic membrane environments. When analyzed in CD, SAAP‐148 did not show defined secondary structure in any of the tested conditions (Figure 1C,D), as the absorption maxima characteristic for a typical α‐helix are not clearly visible in the presence of SDS, DPC, or POPG. In the presence of POPC, no changes to control sample in the presence of Hepes were observed. CD spectra for an α‐helix are characterized by the positive band at 192 nm and negative bands at 208 and 222 nm, which are visible in case of DPC and to a less extent in the case of SDS and POPG. Interestingly, SAAP‐148 showed a shift in the absorbance to 200 nm and a pronounced CD shoulder around 229 nm. These CD extrema between 225 and 230 nm are also observed in other proteins like oxytocin,^[^ ^30^ ^]^ vasopressin,^[^ ^30^ ^]^ fibronectin,^[^ ^31^ ^]^ or in snake venom toxins.^[^ ^32^ ^]^ These observations can be attributed to aromatic side chain interactions.^[^ ^30^, ^32^ ^]^ Such interactions can be assigned to aromatic residues W5, W16, and F9, which when SAAP‐148 folds into α‐helix are predicted to form a hydrophobic core (Figure 1C,D).

### 2.2 SAAP‐148 Permeabilizes Both, Anionic and Zwitterionic Membranes

**Figure 2:**
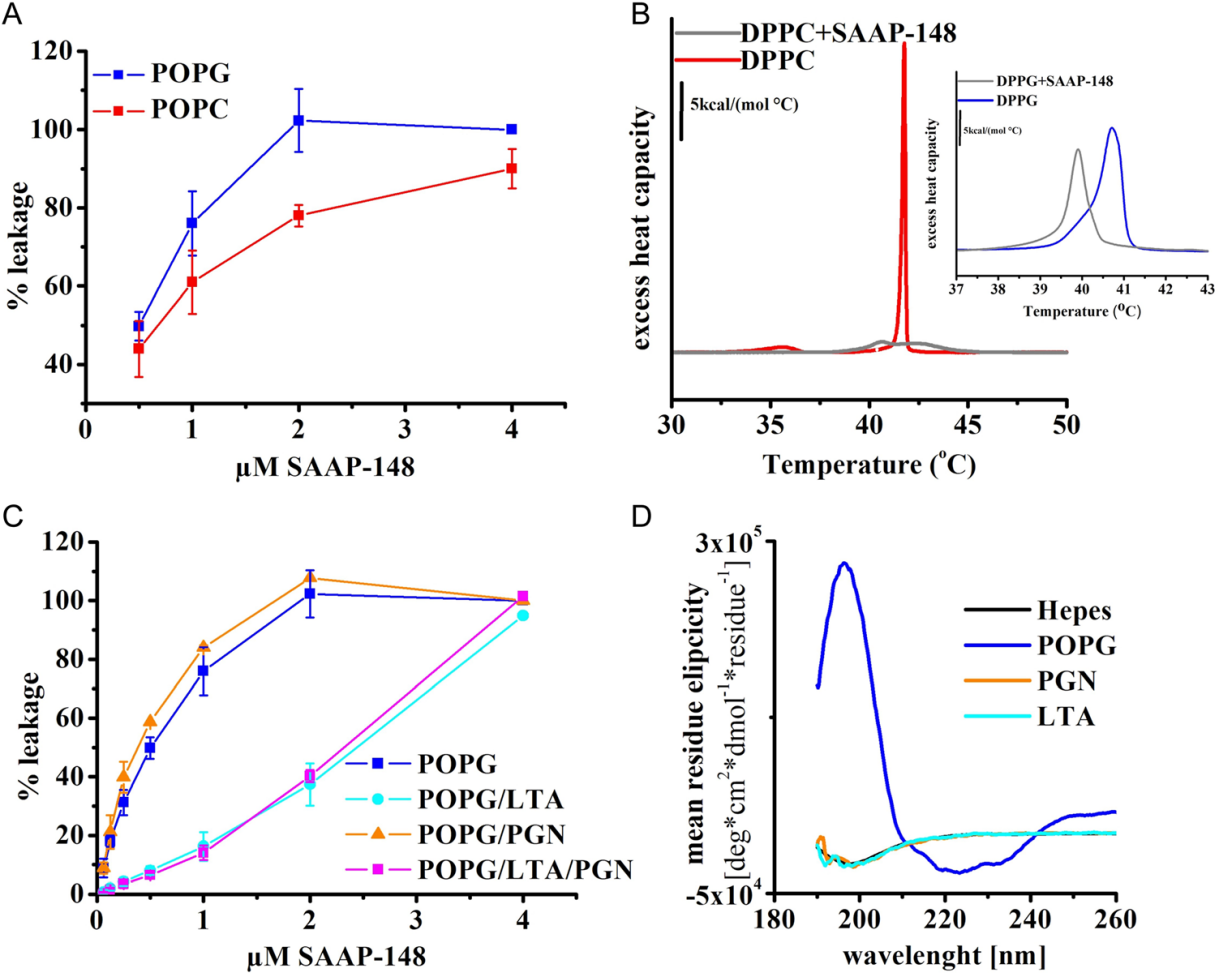
Membrane disordering effect of SAAP‐148. A) Membrane permeability of SAAP‐148 as observed by leakage of POPG (blue) and POPC (red) LUVs induced by SAAP‐148. Data are from at least three independent experiments. Results are means and standard deviations. B) DSC graphs showing thermotropic behavior of DPPC and DPPG (inset) in presence and absence of SAAP‐148. C) Membrane permeability of SAAP‐148 as observed by leakage of POPG (blue) and POPG supplemented with LTA (cyan), PGN (orange), or both LTA and PGN (magenta). Data are from at least three independent experiments. Results are means and standard deviations. D) CD spectra of 200 μm peptide in 10 mm Hepes and in the presence of 2.5 mm POPG (blue), 0.1 wt% peptidoglycan (orange), and 100 μg mL^−1^ LTA (cyan).

Given that SAAP‐48 adopts a well‐defined secondary structure only in the presence of POPG, we wanted to investigate whether this secondary structure impacts the membrane‐disrupting activity of SAAP‐48. In our previous experiments, we showed membrane perturbation effects of SAAP‐148 on highly anionic POPG membranes and partly anionic membranes mainly composed of zwitterionic phosphatidylethanolamine (PE), for example, POPG/POPE and *E. coli* polar lipid extracts.^[^ ^1^, ^21^, ^22^ ^]^ In order to investigate if SAAP‐148 can impact the membrane integrity of solely zwitterionic membranes we performed differential scanning calorimetry (DSC) and leakage experiments. The data obtained imply that SAAP‐148 does permeabilize POPC nearly as strong as it permeabilizes POPG membranes. The release of ANTS, entrapped fluorescent dye, from anionic POPG vesicles was complete at 2 μm SAAP‐148, where from POPC vesicles it reached ≈70% (**Figure** **2**A). In addition, DSC revealed also a strong influence of SAAP‐148 on DPPC phase transition behavior (Figure 2B). The pretransition peak, which is particularly sensitive to insertion of peptides into the bilayer, disappears upon exposure to SAAP‐148 and main transition enthalpies were significantly lower indicating that the peptide strongly disorders DPPC bilayers. Furthermore, SAAP‐148 induced a splitting of the main transition into two smaller, but much broader peaks (with much higher Δ*T*_1/2_ values indicating loss of cooperativity), one below and the second above the main transition of pure DPPC. The upper peak probably corresponds to domains, which indicate tighter packing of lipids that might result either from surface charge shielding, dehydration of the lipid head groups, or induction of specific gel phases such as interdigitation or disaggregation of the multilamellar vesicles (MLVs) into smaller vesicles. The phase transition temperature of DPPC was shown to be dependent on a number of parameters, not only on the rigidity but also on lamellarity, size, and shape of the lipid aggregates.^[^ ^33^ ^]^ Thus higher phase transition temperatures were reported for smaller unilamellar vesicles, although the packing of the lipids in the gel phase of such lipid aggregates is more similar to that of the fluid phase. This was attributed to curvature‐induced decreased lateral packing as indicated by the lower phase transition enthalpy,^[^ ^34^, ^35^ ^]^ which we also observed for SAAP‐148/DPPC. Thus, in the presence of the peptide the MLVs may disaggregate into smaller vesicles with less lipid layers. Alternatively, as demonstrated for the parent peptides LL‐37 and OP‐145, disk‐shaped lipid‐peptide aggregates wrapped by a peptide ring as modeled from small‐angle X‐ray scattering (SAXS) may be formed.^[^ ^36^, ^37^ ^]^ In contrast to the parent peptides, this phase transition was much more pronounced in the case of SAAP‐148. The lower phase transition accompanied with low enthalpic values indicate chain melting and lipid disordering. This also was the case for DPPG membranes (Figure 2B, inset), whereby characteristic thermodynamic parameters were indicative for induction of quasi‐interdigitated structure resulting in membrane thinning and permeabilization.^[^ ^1^ ^]^

As a control to determine whether SAAP‐148's structural formation is impacted by interaction with other bacterial components, we performed CD and leakage experiments in the presence of bacterial cell wall components, lipoteichoic acid (LTA) and peptidoglycan (PGN), as these are the major components of the *S. aureus* membrane, for which SAAP‐148 shows the most promising activity. Although membrane activity is impaired in the presence of LTA (Figure 2C), CD spectra show no secondary structure development in the presence of any of the bacterial cell wall components (Figure 2D). However, the release of the entrapped fluorescent dye was very slow from LTA‐containing vesicles, suggesting that reduced penetration due to aggregates, rather than the ability of the peptide to disrupt POPG vesicles in the presence of LTA, are responsible. This is confirmed by the observation that complete leakage from LTA‐containing vesicles occurs at 4 μm, as compared to 2 μm for pure POPG vesicles.

### 2.3 SAAP‐148 is Partly Helical in Membranes

**Figure 3:**
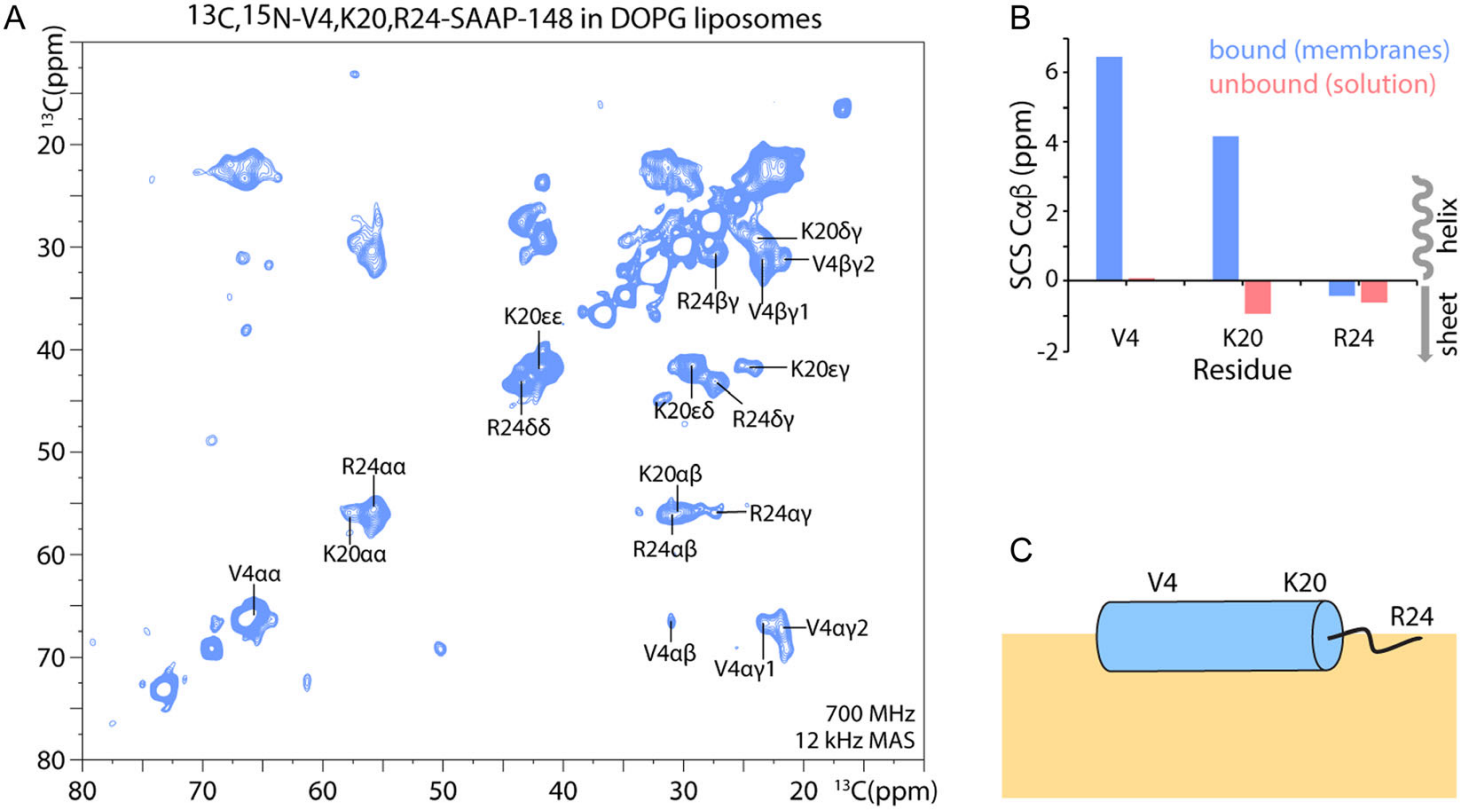
ssNMR of SAAP‐148 in anionic membranes. A) Dipolar‐based 2 D CC PARIS^[^ ^75^ ^]^ spectrum of SAAP‐148 in DOPG. B) SCS of SAAP‐148 in absence and presence of DOPG. C) Scheme of SAAP‐148 insertion in the membrane. Note that the orientation of the peptide could also be transmembrane.

We then used solid‐state NMR (ssNMR) to gauge information on the structure of SAAP‐148 in its membrane‐bound state (**Figure** **3**). To make SAAP‐148 amenable to ssNMR studies, we synthetically introduced three specifically ^13^C‐,^15^N‐labeled residues in the N‐terminus (V4), the C‐terminal region (K20), and the end of the C‐terminus (R24). Next, we incubated SAAP‐148 with hydrated DOPG liposomes and acquired a dipolar‐based 2D CC PARIS spectrum that we could fully assign. From these assignments we calculated so‐called secondary chemical shifts (SCS) which are sensitive reporters on the secondary structure. While SAAP‐148 is unstructured in solution, SCS ssNMR data clearly showed that the peptide changes its conformation upon membrane binding. Here, residues V4 and K20 showed that the N‐terminus and the middle‐to‐C‐terminal region adopt helical conformation, while the C‐terminus adopts no secondary structure.

### 2.4 SAAP‐148 Oligomerizes in Solution and on Membranes

The indication of a potential hydrophobic core prompted us to investigate the aggregation behavior of SAAP‐148 both in solution and on membranes. First, we performed a prediction analysis using AlphaFold.^[^ ^38^ ^]^

**Figure 4:**
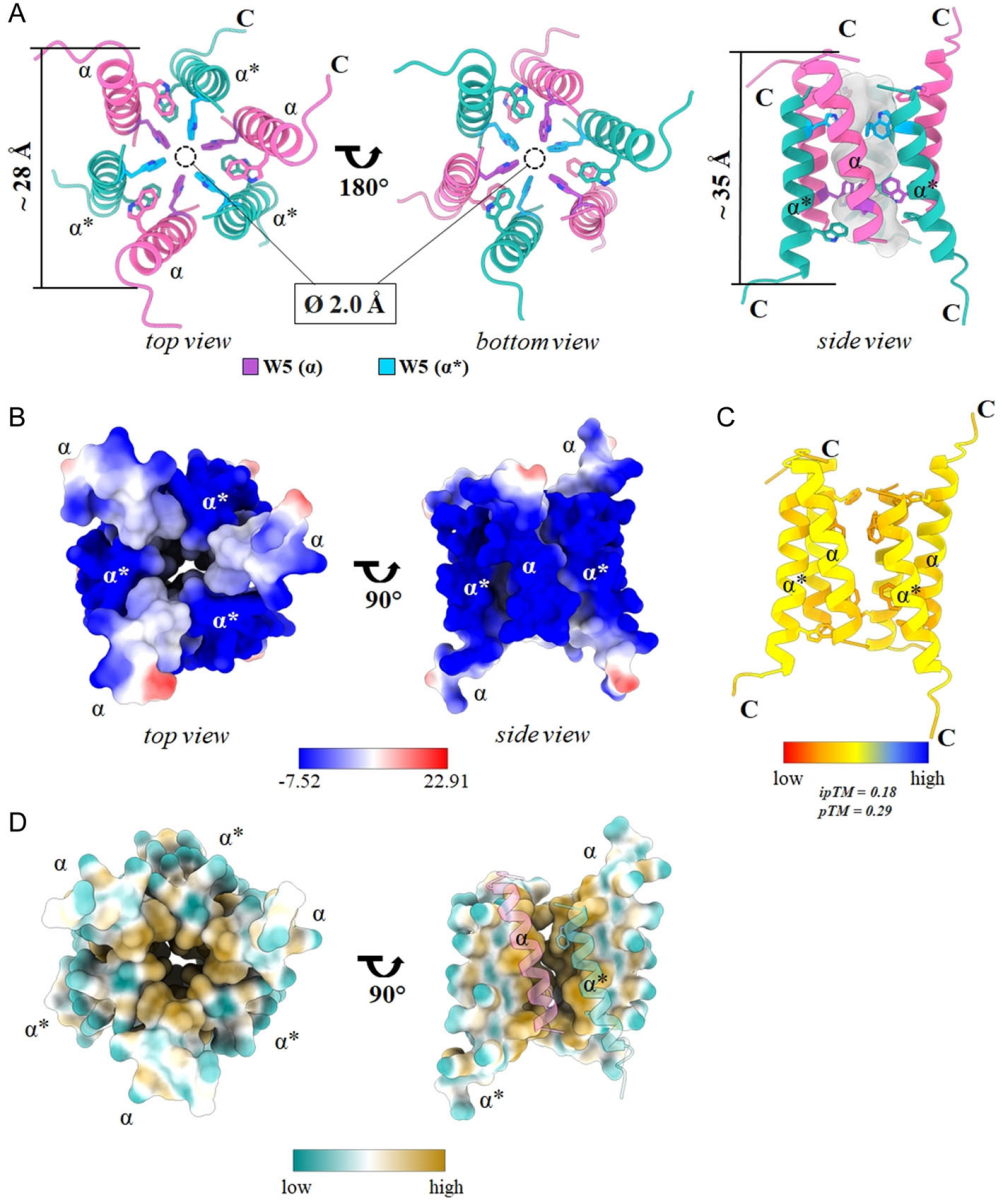
AlphaFold 3 prediction of a SAAP‐148 homo‐hexamer. A) The multimer exhibits a symmetric barrel‐stave‐like structure with W residues 5 and 16 pointing toward the hydrophobic tunnel core forming a 2.0 Å radius tunnel. B) Surface representation colored by electrostatic potential, blue: negative, red: positive. C) Cartoon representation colored by plDTT value. Values range from 38.7 to 76.2. D) Surface representation colored by lipophilicity, blue: lipophobic, yellow: lipophilic.

The predicted oligomeric structure of SAAP‐148 (**Figure** **4**) exhibits a channel architecture comprising a hexameric bundle, formed by ≈35 Å‐long alpha helices. The barrel‐stave‐like channel formation involves dimerization of the parallel peptide trimers resulting in a firmly enclosed channel structure. Calculations reveal an ≈2.0 Å diameter entrance from both sides of the barrel built up by three W5 residues of every other SAAP‐148 monomer (Figure 4A). The theoretical radius of gyration was calculated to be 17.71 Å. The barrel exhibits a highly lipophilic core on the inside as well as a negative electrostatic potential on the hydrophilic outside (Figure 4B,D).

**Figure 5:**
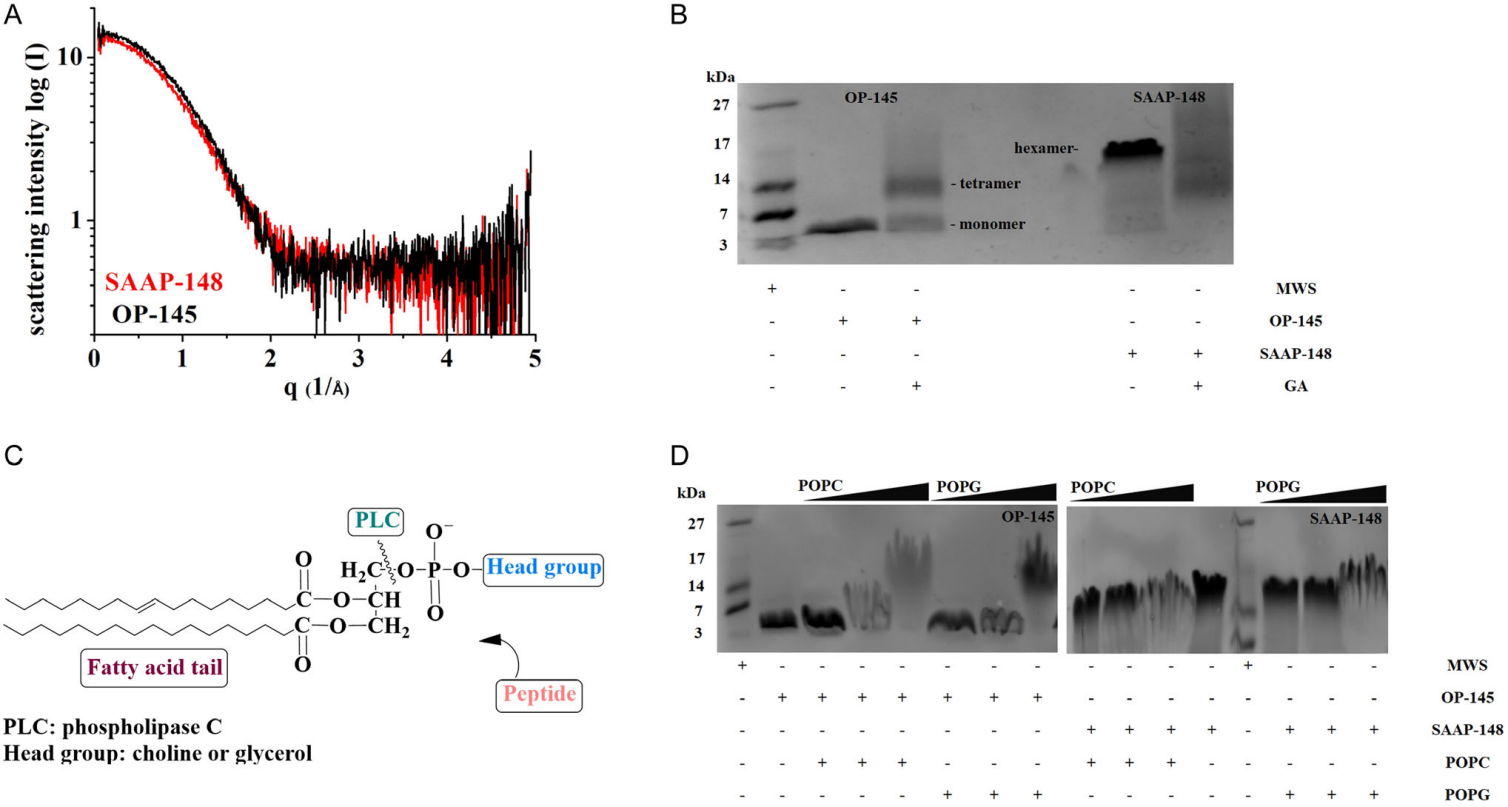
Aggregation of SAAP‐148 in solution and on membranes A) SAXS Measurements performed in a solution of the OP‐145 or SAAP‐148 dissolved (*c* = 2 mg mL^−1^) in 20 mm NaPi/120 mm NaCl‐buffer (pH = 7.0). OP‐145, which is also a 24‐mer peptide, was used as a control. Both curves coincide over the calculated *q*‐range. The radius of gyration 16.1 ± 0.2 Å for OP‐145 and 16.6 ± 1 Å for SAAP‐148. B) Detection of SAAP‐148 aggregates in Hepes by 18% SDS‐PAGE. OP‐145, which has nearly the same molecular weight was used as control. Peptide were tested in presence and absence of crosslinking reagent glutaral aldehyde (0.2 v%). C) Schematic illustration of phospholipid cleavage by PLC. D) SDS‐PAGE of SAAP‐148 after incubation with POPG and POPC at lipid‐to‐peptide molar ratio of 0/100/25:1 following the sequential lipid degradation by 0.3 unit mL^−1^ phospholipase (D). Ultralow molecular weight standard was used as a molecular weight marker. Data are from a representative experiment of at least three independent experiments.

Additionally, we performed SAXS measurements on a solution of peptides (*c* = 2 mg mL^−1^) SAAP‐148 and OP‐145 dissolved in 20 mm NaPi/120 mm NaCl‐buffer (pH = 7.0). OP‐145 was used as it is a linear alpha helical peptide that has the same length of 24 amino acid residues (**Figure** **5**A). From the ± SAXS curves we can obtain information about size, shape, and symmetry. The size is represented by the parameter *R*_g_, the so‐called radius of gyration. For both OP‐145 and SAAP‐148 we obtained *R*_g_ of around 16 Å. The expected radius of gyration measured with SAXS for a helical 24‐mer peptide, as it is OP‐145 and SAAP‐148, is about 10–11 Å [Radius of gyration can be calculated using following equation$\sqrt{{\frac{r}{2}}^{2}+{\frac{\frac{l}{2}}{3}}^{2}}$ estimated to be 10.39 Å for 24 amino acid‐long SAAP‐148 (3.6 amino acid pro helix turn of 5.4 Å = 24 × 5.4/3.6 is about 36 Å long with helix diameter of 20 Å) and is much smaller than the experimental 16 Å we obtained from our measurements]. Of note, this calculation is based on the assumption of 100% helical content. It is noteworthy that the *R*_g_ of 16 Å for SAAP‐148 is slightly smaller than the hydrodynamic radius of 15 Å, which was estimated by pulse‐field NMR. However, larger *R*_g_ values indicate that the shape of the peptide structure may slightly deviate from the globular structures.^[^ ^39^ ^]^ This deviation is also reflected by the *R*_g_/*R*_h_ quotient of >1, typical globular proteins exhibit an *R*_g_/*R*_h_ quotient of 0.775.^[^ ^40^ ^]^ In agreement with SAXS and AlphaFold modeling, SDS‐polyacrylamide gelelectrophoresis (SDS‐PAGE) analysis of SAAP‐148 revealed a clear band at ≈18 kDa, which corresponds to the formation of a hexameric peptide (Figure 5B). Interestingly, OP‐145 was detected at 3 kDa, which corresponds to its monomeric form. However, in the presence of crosslinking reagent glutaral aldehyde, a heteromeric fraction of monomeric, dimeric, and tetrameric OP‐145 was detected, indicating OP‐145`s tendency for oligomerization. Of note, in the presence of crosslinking reagent we did not detect significant bands in case of SAAP‐148 as higher aggregates most likely prevent peptides mobility through the SDS gel. Although glutaral aldehyde primarily crosslinks lysins residues in a peptide, the caution should be exercised in interpretation as it may also nonspecifically crosslink lysins or other residues that do not remain in close proximity to one other.^[^ ^41^ ^]^

Given the fact that SDS‐PAGE typically detects peptides/proteins in their reduced, unfolded form, the observation of oligomerized SAAP‐148 suggests that the peptide forms a highly stable aggregate structure. To explore whether SAAP‐148 disaggregates upon interaction with membranes, we incubated the peptide with increasing concentrations of POPG/POPC and performed SDS‐PAGE. However, detecting the peptide bands was challenging due to increased smearing, indicating strong interactions between the peptide and lipid molecules. To resolve this, we treated the peptide/lipid mixtures with phospholipase C, which cleaves the phospholipid backbone at the site just before the phosphate group, leaving the headgroup glycerol and phosphate intact and bound to the peptides (Figure 5C). This treatment allowed for the clear detection of peptide bands in SDS‐PAGE (Figure 5D). In both the presence and absence of membranes, SAAP‐148 was detected as a hexameric peptide. No other peptide bands were observed, suggesting that SAAP‐148 remains in its aggregated form during interaction with membranes. In contrast, the aggregation pattern of OP‐145 was different. As the lipid concentration increased, bands corresponding to higher molecular weights appeared, indicating that OP‐145 tends to aggregate upon membrane interaction. These aggregates are less stable compared to those of SAAP‐148. Interestingly, no significant difference in aggregation status was observed between POPC and POPG membranes for the two peptides.

### 2.5 MD Simulations Indicate SAAP‐148 Oligomerization on the Membrane Interface

**Figure 6:**
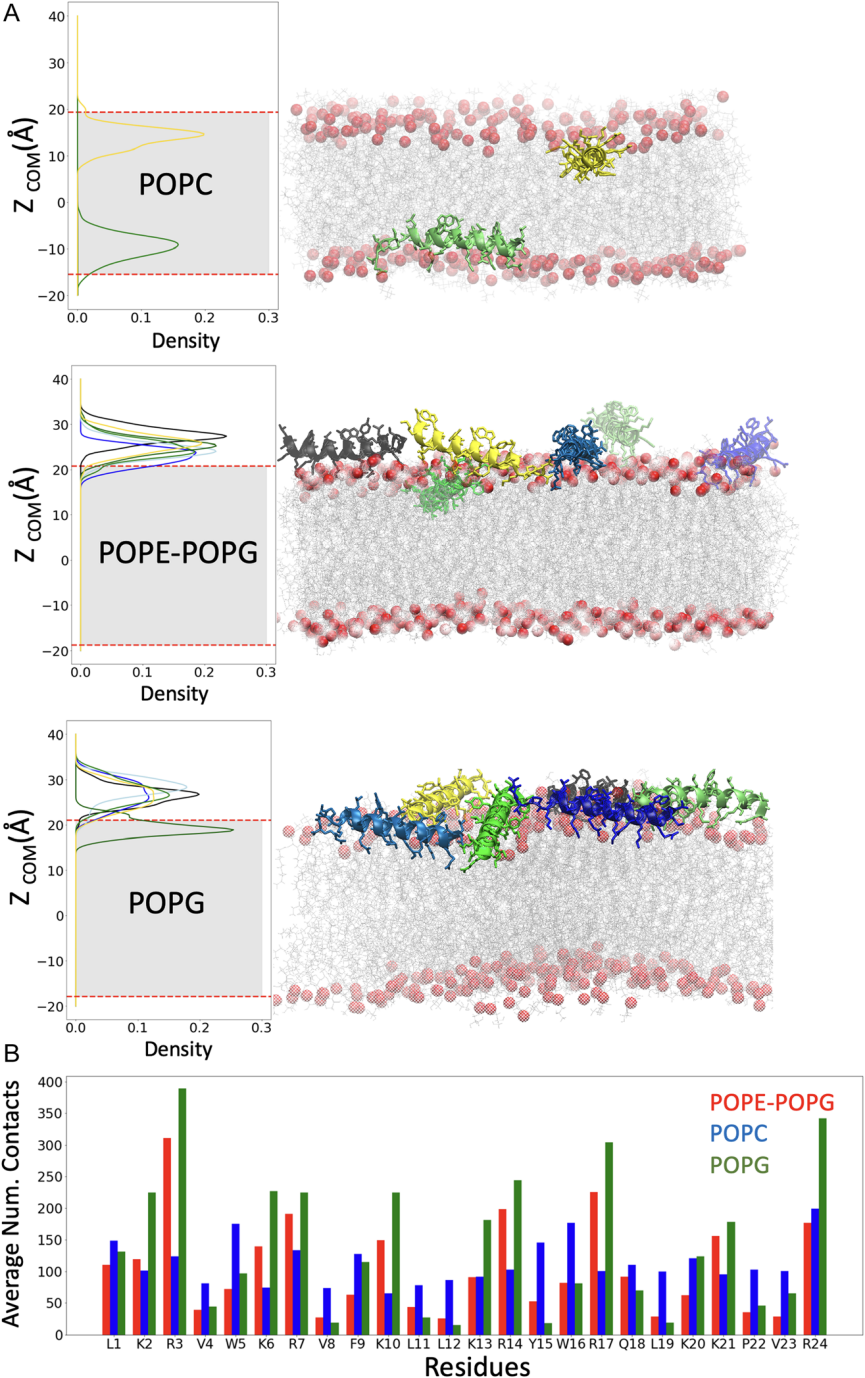
MD simulation of SAAP‐148 interactions with membranes A) Density distribution of the z‐component of COM between SAAP‐148 computed over the last 200 ns of the MD simulation, in relation to the average z‐components of the phosphate positions of the POPC, POPE/POPG, and POPG bilayers depicted as broken red lines. On the right, the last snapshot of the corresponding MD simulation is illustrated, representing the SAAP‐148 as cartoons and the phosphate head groups as red spheres. B) Average number of contacts between SAAP‐148 and POPG, POPE/POPG, and POPC membranes, respectively. The contact is established when the distance between atoms of the two group is less than 4.5 Å.

Molecular dynamics (MD) simulations of SAAP‐148 (**Figure** **6**) were conducted in the presence of membranes of varying composition, including zwitterionic POPC (eukaryotic model), anionic POPG (Gram‐positive bacterial model), and a POPE/POPG mixture in a 3:1 molar ratio (Gram negative bacterial model). The estimated membrane thicknesses were 39.6 ± 0.2 Å for POPG, 38.9 ± 0.1 Å for the POPE/POPG mixture, and 34.8 ± 0.2 Å for POPC, which corresponded well with experimentally measured phosphor–phosphor distances for these bilayers.^[^ ^42^ ^]^ The peptides were initially positioned 10 Å above the membranes. Within the first picosecond of the MD simulation, SAAP‐148 began interacting with the anionic glycerol head groups of the POPG and POPE‐POPG membranes via its C‐terminal arginine side chain. This interaction progressively strengthened as the simulation progressed. The average number of contacts between the heavy atoms, that means between all atoms except for hydrogens of SAAP‐148 and membranes, was calculated and averaged over the final 200 ns of the simulations. As expected, the strongest interactions were observed between SAAP‐148 and the pure POPG membrane (Figure 6B). These interactions were primarily facilitated by the positively charged arginine and lysine residues in SAAP‐148, with R3 making the most significant contribution. The peptides seem to remain on the surface of the membrane forming fibril‐like structures as depicted in Figure 6A for the POPG and POPE–POPG membranes. Interesting is the interaction of SAAP‐148 with the POPC lipids. Among the six peptides initially positioned near the POPC membrane, only two successfully reach the membrane surface, while the remaining four drift away (Figure 6A). The zwitterionic nature of the POPC headgroup offers weaker attraction to the positively charged peptides compared to the stronger interactions observed with POPG. Nonetheless, once SAAP‐148 reaches the membrane surface, it demonstrates stronger interactions with the POPC headgroups and penetrates deeper into the bilayer than it does with POPG. The density profiles analyzed over the final 200 ns of the MD simulation (Figure 6A) reveal that SAAP‐148 aligns parallel to the membrane surface, with its center of mass (COM) positioned 5 Å beneath the phosphate layer of POPC. Due to the limited number of peptide molecules reaching the surface and their immediate penetration into the membrane upon contact, no peptide aggregation was observed.

## 3 Discussion and Conclusions

In general, it is widely recognized that the basic physicochemical properties that characterize a peptide as an AMP are cationicity, hydrophobicity and amphipathicity.^[^ ^43^, ^44^, ^45^ ^]^ When designing novel peptides, these parameters are typically considered, as they are assumed to primarily determine the peptides’ interaction with bacterial membranes. The charge is important for the recognition of bacteria, traversing the bacterial cell wall and interacting with negatively charged lipid components on the bacterial surface, such as lipopolysaccharides (LPS), (lipo)‐teichoic acids, and phospholipids like PG or cardiolipin. Hydrophobicity is crucial for insertion into the hydrophobic core of the membrane. Peptides with larger hydrophobic areas are generally thought to disrupt membranes more efficiently. For instance, SAAP‐148 has a more extensive hydrophobic surface compared to its parent peptide OP‐145 and indeed shows much stronger interactions with membranes, leading to greater disruption of membrane integrity both at the molecular and cellular level.^[^ ^20^, ^21^, ^22^ ^]^ However, this comes with a drawback: peptides with larger hydrophobic faces can also insert into and damage eukaryotic membranes. This is reflected in the increased cytotoxicity of SAAP‐148 toward eukaryotic cells compared to OP‐145.^[^ ^36^, ^46^ ^]^ Although bacterial and eukaryotic membranes differ in composition, it appears that SAAP‐148 cannot significantly distinguish between Gram‐positive and Gram‐negative bacteria and human cells. It exerts antimicrobial activity against a variety of bacterial strains at similar concentrations, where it also shows cytotoxicity to human cells.^[^ ^1^, ^46^ ^]^

When we compare the interaction of SAAP‐148 at the molecular level, especially with membranes composed of anionic POPG (a major component of bacterial membranes) and membranes composed of zwitterionic POPC (found in the outer leaflet of eukaryotic membranes), we do not observe a significant difference. SAAP‐148 interacts with both types of membranes (Figure 1, 2, 5, 6), resulting in membrane disruption for both (Figure 2A). The only noticeable difference is the penetration depth into the hydrophobic core of the POPC and POPG bilayers. MD simulations clearly show that SAAP‐148 molecules penetrate below the POPC head groups, while they remain on the membrane surface in the case of POPG (Figure 6). The interaction between the cationic peptide residues (*R* and *K*) and the phosphate groups of POPG helps keep the peptide on the surface (Figure 6). Indeed, zetapotential measurements observed in our earlier studies indicate that SAAP‐148 overcompensates the surface charge of charged membranes including *E. coli* lipid vesicles.^[^ ^21^ ^]^ This is likely one of the reasons why the determination of the secondary structure in the presence of anionic and zwitterionic micelles and liposomes caused some discrepancies. The alpha‐helical structure of the peptide cannot be detected when it penetrates into the hydrophobic core. The hydrodynamic radius of the DPC micelles did not change in the presence of SAAP‐148 while the peptide increased the hydrodynamic radius of SDS micelles, suggesting that SAAP‐148 is entrapped in the DPC micelles. However, the aggregation of SAAP‐148 may be the primary reason for measurement inconsistencies in some methods.

The prediction analysis using AlphaFold3 clearly shows the formation of a hexameric SAAP‐148 structure, bundled in a channel‐like formation of antiparallel dipeptide trimers, held together by aromatic residues at positions W5, W16, and F9 (Figure 4). This is in line with the detected signals for hydrophobic core formation in our CD measurements (Figure 1) and with stable bands of higher molecular weight in SDS‐PAGE (Figure 5B). The bands observed around 18 kDa likely correspond to six SAAP‐148 molecules of ≈3 kDa each. Even X‐ray scattering and pulsed‐field NMR measurements indicated that SAAP‐148 might form oligomers, as the calculated radius of gyration and hydration were not consistent with a single‐SAAP‐148 molecule (Figure 5A). Interestingly, a channel‐like tetrameric structure of LL‐37 was also described in the presence of membrane mimics.^[^ ^47^ ^]^ However, a highly charged inner core was detected. Although MD simulations demonstrated pathways for the passage of water molecules and small ions, the authors concluded that channel formation is necessary to increase the local concentration of the peptide on the membrane surface and to disrupt the transmembrane potential to kill the bacterium. In the presence of membrane mimics, SAAP‐148 most likely retains its hexameric channel‐like structure, as we did not observe the monomeric peptide in SDS‐PAGE experiments after incubation with either POPC or POPG membranes (Figure 5). This was not the case for OP‐145, which, due to the absence of aromatic residues, does not form a stable oligomer and disaggregates from the membrane surface once the interaction becomes stronger (Figure 5). It is worth noting that OP‐145 forms an ideal alpha helix in the presence of membrane mimics.^[^ ^36^ ^]^ However, higher degrees of oligomerization were detected in X‐ray and SDS‐PAGE experiments (Figure 5). Also for other AMPs, different degrees of oligomerization were observed in different membrane‐mimicking environments^[^ ^48^ ^]^ including temporins,^[^ ^49^ ^]^ protegrins,^[^ ^50^ ^]^ melittin,^[^ ^51^ ^]^ with various methods being used. While it is commonly postulated that peptides oligomerize within the membrane, aligning in specific patterns to disrupt membrane integrity, as described in classical carpet or pore models, some peptides may self‐assemble into oligomers prior to reaching the membrane.^[^ ^52^, ^53^ ^]^ These preoligomers may affect binding to and insertion into the membrane, potentially leading to faster or more extensive disruption.^[^ ^48^, ^53^ ^]^

The differences between OP‐145 and SAAP‐148 led us to assume that SAAP‐148 forms a very stable oligomeric structure. Interestingly, MD simulations indicated increased oligomerization of SAAP‐148 on the membrane surface, and in the presence of anionic POPG, the peptide molecules formed a fibril‐like structure (Figure 6). The self‐assembly of misfolded proteins or peptides into highly organized fibrillar morphology is characteristic for amyloids known to induce neurodegenerative disorders.^[^ ^54^, ^55^ ^]^ The formation of fibril‐like structures is commonly observed in AMPs^[^ ^48^ ^]^ and peptides with conformationally flexible structures that form unstable oligomers (such as LL‐37 or OP‐145) might, through external stimuli or side‐chain interactions with lipids, transition from a lower oligomeric state to fibril.^[^ ^47^ ^]^ It is not sure whether SAAP‐148, like LL‐37 of OP‐145, forms fibril structures on the surface of all types of membranes since MD simulation studies of SAAP‐148 in the presence of a membrane are not conclusive. It is plausible that the peptide, in its oligomeric form stabilized by aromatic residues in solution, could expose positively charged side‐chain residues on its surface, enabling fibril formation on anionic membranes. In regard to the mode of action at the cellular level, the first contact of the oligomeric peptide with the bacterial surface results in increased interaction with the highly charged cell wall components, LTA and LPS. This, in turn, leads to a lower local peptide concentration and reduced activity on the membrane, as demonstrated in Figure 2. The presence of LTA in POPG vesicles leads to slower penetration of the peptide and less membrane leakage compared to POPG alone. Most importantly, different environments, including solvents^[^ ^21^ ^]^ and the bacterial surface,^[^ ^20^ ^]^ might influence the degree of oligomerization, which in turn could impact the peptide's activity against diverse resistant strains, particularly those with more complex membrane envelopes, such as multidrug‐resistant *S. aureus* or *Klebsiella pneumoniae*. AMPs Magain‐2 and PGLa were observed to form fibrils in Müller–Hinton, which led to their reduced antimicrobial activity against *E.coli* but the fibril formation did not influence the synergistic enhancement of activity of the equimolar mixture of both peptides.^[^ ^56^ ^]^ When comparing SAAP‐148's antimicrobial activity against Gram‐positive strains like *Enterococcus hirae*,^[^ ^22^ ^]^ multidrug‐resistant *Enterococcus fecium,* or *S. aureus*,^[^ ^1^ ^]^ the bactericidal concentration shifts from 0.4 to 1.6 μm. This trend is also observed against Gram‐negative bacteria, where the bactericidal concentration against *E. coli* is about 0.8 μm, and against *K. pneumoniae* or *Enterobacter cloacae*, it ranges from 6.4 to 12.8 μm.^[^ ^1^ ^]^ The more complex the membrane architecture, the higher the amount of peptide required to kill the bacterium. In our previous study, we also observed a 2‐ to 4‐fold improvement in bactericidal activity against *E. coli* strains lacking certain components of the LPS, which might also point to facilitated penetration of the aggregates.^[^ ^20^ ^]^ In fact, SAAP‐148 is one of the most effective AMPs, exhibiting activity across various bacteria with diverse membrane architectures. This potency is likely attributed to its overall structural properties, discussed previously in our studies^[^ ^1^, ^20^, ^21^ ^]^ and here with regard to oligomerization.

In summary, our previous studies demonstrated the interaction of SAAP‐148 with bacteria and outlined its mode of action in terms of its target molecules and the basic physicochemical properties that lead to these interactions and bacterial killing. In this article, we primarily focused on elucidating the peptide's secondary structure. To achieve this, we employed a series of commonly used biophysical techniques, including NMR, CD, and X‐ray scattering methods, as well as simulation studies like AlphaFold3 and MD simulations. In nearly all of these studies, we identified SAAP‐148 in an aggregated state, which led to difficulties in detecting it as a helical peptide. However, in all cases, evidence for the formation of an alpha helix was present, demonstrating that SAAP‐148 forms a stable hexamer that creates a hydrophobic core via aromatic residues both in solution and on the membrane.

## 4 Experimental Section

4.1

4.1.1

##### Chemicals

OP‐145 (acetyl‐IGKEFKRIVERIKRFLRELVRPLR‐amide) and SAAP‐148 (acetyl‐LKRVWKRVFKLLKRYWRQLKKPVR‐amide) were synthesized as previously described.^[^ ^20^, ^22^ ^]^ Additionally, SAAP‐148 was synthesized using ^13^C,^15^N‐labeled residues in the N‐terminus (V4), the C‐terminal region (K20), and the end of the C‐terminus (R24) following the same procedure for peptide synthesis. The purity of the peptides, determined by ultra performance liquid chromatogryphy‐mass spectroscopy (UPLC‐MS) (Acquity, Waters, Milford, MA), was >95%, their identity was confirmed with matrix assisted laser desorption ionization‐time of flight (MALDI‐TOF) mass spectrometry (Microflex, Bruker, Bremen, Germany). The peptides were stored at −20 °C until use and then dissolved in 0.1% acetic acid pH 3.3 to a stock of 10 mg mL^−1^. Aliquots were also stored at −20 °C. All phospholipids (>99% purity) were obtained from Avanti Polar Lipids (Alabaster, AL) and stored at −20 °C before usage. The synthetic phospholipids used in this study were DPPG (1,2‐dipalmitoyl‐sn‐glycero‐3‐[phospho‐rac‐(1‐glycerol)]), DPPC (1,2‐dipalmitoyl‐sn‐glycero‐3‐[phospho‐rac‐(1‐glycerol)]), POPG (1‐palmitoyl‐2‐oleoyl‐sn‐glycero‐3‐[phospho‐rac‐(1‐glycerol)], POPC (1‐palmitoyl‐2‐oleoyl‐sn‐glycero‐3‐phosphatidylcholine), 1,2‐dioleoyl‐sn‐glycero‐3‐phosphocholine (DOPC), and 1,2‐dioleoyl‐sn‐glycero‐3‐phosphoglycerol (DOPG). The deuterated sodium dodecyl sulfate (SDS‐d_25_ 98%) and the dodecylphosphocholine (DPC‐d_38_ 98%) were purchased from Cambridge Isotope Laboratories Inc. (Andover, MA) and deuteriumoxide D_2_O from euriso‐top with a purity of 99.9%.

##### Liposome Preparation

Preparation of liposomes was conducted according to previously published protocols.^[^ ^22^ ^]^ For DSC analysis, lipid films were prepared by evaporating 1 mg of lipids from 10 mg mL^−1^ stock solutions of individual lipids under nitrogen stream, followed by overnight vacuum drying. The resulting lipid films were stored at 4 °C until use. Lipid vesicles were formed by adding phosphate buffered saline (PBS) (20 mm NaH_2_PO_4_/Na_2_HPO_4_, 130 mm NaCl, PH = 7.4) to the lipid films. For peptide‐containing vesicles, peptides were dissolved in PBS to a final volume of 1 mL (resulting in a concentration of 1 mg mL^−1^) before being added to the lipid films. Vesicle formation was induced by intermittent vigorous vortexing, with the samples incubated at temperatures above the phase transition temperatures of the respective phospholipids. Specifically, the samples were placed in a sand bath at 65 °C for DPPG and 50 °C for DPPC, while POPC and POPG were performed at the room temperature and incubated for 1–2 h.

For leakage measurements, liposomes with a concentration of 20 mg mL^−1^ in fluorophore buffer (10 mm HEPES, 68 mm NaCl, 12.5 mm ANTS, 45 mm DPX, pH 7.4) were used. Large unilamellar vesicles (LUVs) of ≈100 nm in size were prepared by extruding the hydrated liposomes through a 0.1 μm polycarbonate filter (Millipore‐Isopore) for 20 cycles. ANTS (8‐aminonaphthalene‐1,3,6‐trisulfonic acid, disodium salt) and DPX (p‐xylene‐bis‐pyridinium bromide) were obtained from molecular probes (Eugene, OR). Vesicle size was determined using a Zetasizer (Zetasizer NANO, Malvern Instruments, Herrenberg, Germany).

For NMR analysis, LUVs were prepared by dissolving corresponding lipid films in 20 mm Tris, 25 mm NaCl, pH 7.4 followed by the extrusion procedure described above.

##### Leakage Measurement of ANTS/DPX‐Loaded Liposomes

Leakage of the aqueous contents from ANTS/DPX‐loaded liposomes composed of POPG, POPG/LTA, POPG/PGN, POPG/LTA/PGN, or POPC upon incubation with SAAP‐148 was assessed following procedures outlined in previous studies.^[^ ^22^ ^]^ In brief, 20 mg mL^−1^ ANTS/DPX‐loaded lipid vesicles of a defined size were separated from free fluorescent dye using exclusion chromatography on a Sephadex G‐75 (Amersham Biosciences) column, swollen in an iso‐osmotic buffer (10 mm HEPES, 140 mm NaCl, pH 7.4). The phospholipid concentration was determined via phosphate analysis as described earlier. Fluorescence emission from the ANTS/DPX‐loaded lipid vesicles was measured at 37 °C with an excitation wavelength of 360 nm and emission at 530 nm, using a slit width of 10 nm for both excitation and emission monochromators. Lipid concentrations of 50 μm were used for the measurements. Fluorescence intensity (IF) was recorded as a function of time before and after the addition of increasing amounts of peptide (ranging from 0.125 to 16 μm), corresponding to peptide‐to‐lipid molar ratios of 1:400 to 1:3. The experiments were conducted on a VARIAN Cary Eclipse fluorescence spectrophotometer, with data analyzed using Cary Eclipse Software (Scan). Leakage percentage was calculated by measuring the fraction of leakage (IF), where the initial fluorescence (*F*_o_) before peptide addition and the maximum fluorescence (*F*_max_) corresponding to 100% leakage (achieved by adding 20 μL of 10% Triton X‐100) were used for normalization.

##### Differential Scanning Experiment (DSC)

DSC measurements were performed using a Microcal VP‐DSC high‐sensitivity differential scanning calorimeter (Microcal, Northampton, MA) as described earlier.^[^ ^22^ ^]^ Scans were conducted at a lipid concentration of 1 mg mL^−1^ with a constant heating rate of 30 °C h^−1^. Data were analyzed using Microcal's Origin software. Calorimetric enthalpies were calculated by integrating the peak areas after baseline correction and normalizing to the mass of phospholipid. The phase transition temperature was defined as the temperature at the peak maximum.

##### NMR

The NMR samples without membrane mimetics were prepared out of 40–60 μL of a peptide in buffer solution (20 mg mL^−1^) and 60 μL D_2_O. HEPES (10 mm Hepes, pH = 7.4) was added to reach an end volume of 600 μL. The samples containing DPC consisted of 40–60 μL of a peptide in buffer solution (20 mg mL^−1^) dissolved in 100 mm DPC‐d_38_ and HEPES buffer including 10% D_2_O. The same procedure was applied involving the SDS‐containing samples with the exception that SDS‐d_25_ had to be 200 mm in the end solution. The final concentration of the peptides in the samples was about 0.4–0.6 mm. A total correlation spectroscopy (TOCSY) and a NOESY spectrum were acquired on the Bruker Avance III 700 MHz spectrometer at 300 K. The TOCSY cross‐peaks were initially assigned to associate the peptide sequence. The 2D TOCSY experiment allows assigning a whole spin system regardless of the exact topology. The TOCSY peak list was then transmitted to the NOESY spectrum which included all the TOCSY crosspeaks and crosspeaks from correlations between protons which were locally close to each other and may belong to different spin systems. The NOESY crosspeaks were assigned manually. All peak assignments were carried out with the application NMRviewJ. Afterward, the intensities and volumes of the selected areas were calculated with the referred program and then translated into distance restraints using the median method. The 334 nuclear overhause effects (NOEs) were then used to calculate the structures with the program CNS. To improve the alpha helical secondary structure element a hydrogen bond constraints file was included in the refinement. A total of 20 accepted structures were calculated and the 10 structures with the lowest energies were chosen to obtain the (RMSD) value. The value defined the correspondence degree between the individual structures and was about 0.34 Å for the area between the amino acids 4 and 20. The terminal regions were not taken into account because they didn't show defined structures.

The 2D PARIS CC ssNMR spectrum was run on a 700 MHz magnet (^1^H frequency) using 12 kHz MAS.

##### Circular Dicroism (CD)

The secondary structure of SAAP‐148 was determined from CD spectra following the same method used for OP‐145, as described in previously published studies.^[^ ^36^ ^]^ In case of LTA and PGN measurements, peptide was mixed with either 20 and 100 μg mL^−1^ LTA or 0.0.1 and 0.1 wt% PGN. The spectra were recorded on a Jasco J‐715 spectropolarimeter (Jasco, Gross‐Umstadt, Germany) using a 0.02 cm quartz cuvette equilibrated at 25 °C. Spectra were collected at 25 °C between 180 and 260 nm with a step resolution of 0.2 nm. Measurements were performed with 200 μm SAAP‐148, both in the presence and absence of LUVs composed of POPG and POPC, at a peptide‐to‐lipid molar ratio of 1:25. The background signal from 10 mm HEPES (pH 7.0) was subtracted from the spectra. Five individual spectra were averaged, and secondary structure content was estimated using the CDSSTR program on the DICHROWEB online server.^[^ ^57^ ^]^

##### SDS‐PAGE and Phospholipase Degradation Assay for Aggregation of the Peptides

In a total volume of 10 μL, 35 mm POPG or POPC in 10 mm HEPES, or 10 mm HEPES alone, was incubated with either OP‐145 or SAAP‐148 at lipid‐to‐peptide ratios of 100:1, 25:1, and 6:1 for 30 min at room temperature. Phospholipid degradation was induced by adding 0.5 μL of Phospholipase C from *Clostridium perfringens* (PLC, 0.3 units mL^−1^, Sigma, Austria) and 1 μL of 50 mm CaCl_2_ (Sigma, Austria), followed by incubation at 37 °C overnight with shaking. Samples were then mixed with loading buffer containing 10% SDS (without reducing agents) and loaded onto an 18% SDS‐PAGE gel. For crosslinking experiments, peptide were mixed with 0.1% glutaral aldehyde and incubated overnight in the dark at 37 °C.

##### Structure Prediction

The multimer structure of SAAP‐148 was performed using AlphaFold3 with standard settings accessed through the AlphaFold3 Beta Server on 11.11.2024.^[^ ^38^ ^]^ Radius of gyration calculation from the predicted model was performed with PyMOL Molecular Graphics System, Version 2.0 Schrödinger, LLC, using gyradius function within the PyMOL Script COllection (psico) module (https://github.com/speleo3/pymol‐psico).

Molecular graphics and analyses were performed with UCSF ChimeraX, developed by the Resource for Biocomputing, Visualization, and Informatics at the University of California, San Francisco, with support from National Institutes of Health R01‐GM129325 and the Office of Cyber Infrastructure and Computational Biology, National Institute of Allergy and Infectious Disease.^[^ ^58^ ^]^

##### X‐Ray Scattering

The SAXS curves of 2 mg mL^−1^ SAAP‐148 dissolved in PBS buffer were recorded at the ESRF BM29 BioSAXS beamline following the previously described technical details.^[^ ^59^, ^60^ ^]^

Radius of gyration was calculated using Atsas software.^[^ ^61^ ^]^

##### MD Simulations

The initial structure of the peptide was modeled using the published structure of LL‐37 (PDB code: 2K6O) as a template.^[^ ^62^ ^]^ The LL‐37 derivative was designed with an acetylated N‐terminus and an amidated C‐terminus, resulting in an overall charge of +11. To replicate the experimental conditions, the protonation states of all ionizable groups were assigned based on a physiological pH of 7. All MD simulations were performed using the NAMD2.9 program and the CHARMM27 force field.^[^ ^63^, ^64^, ^65^, ^66^ ^]^ A two‐step protocol was applied to solvate and ionize the model system using VMD software plug‐ins:^[^ ^67^ ^]^ first, the peptide was solvated in a cuboid box of TIP3P water molecules, and then a sufficient number of NaCl ions were added to achieve an ionic strength of 130 mm.^[^ ^68^ ^]^ The conventional all‐atom MD simulation began with 20,000 steps of energy minimization using the conjugate gradient algorithm, followed by thermal equilibration at 310 K. During equilibration, heavy atoms were progressively unconstrained.

The equilibrated peptide in aqueous solution was simulated for 100 ns at 310 K under constant particle number (*N*), pressure (*P*), and temperature (*T*) conditions. Pressure was maintained at 1.01325 bar using the Langevin piston method^[^ ^69^ ^]^ with a period of 200 fs and a decay time of 100 fs. To ensure stable pressure regulation, the piston was coupled to a heat bath at 310 K. The production run was conducted under periodic boundary conditions with extended electrostatics via particle mesh Ewald summation and a cut‐off distance of 12 Å for Van der Waals interactions. Bond lengths between heavy and hydrogen atoms were constrained using the SHAKE algorithm, allowing for a 2 fs time step.^[^ ^70^, ^71^ ^]^ The peptide structure obtained after 100 ns of simulation served as the starting configuration for developing model systems that included lipid membranes. Three distinct model membranes were constructed in CHARMM‐GUI, based on the experimental setup: 1) a mixed lipid membrane consisting of 1‐palmitoyl‐2‐oleoyl‐sn‐glycero‐3‐phosphoethanolamine (POPE) and 1‐palmitoyl‐2‐oleoyl‐sn‐glycero‐3‐phosphoglycerol (POPG) in a 3:1 ratio, mimicking a Gram‐negative membrane; 2) a pure POPG membrane, representing a Gram‐positive bacterial surface; and 3) a membrane composed solely of 1‐palmitoyl‐2‐oleoyl‐sn‐glycero‐3‐phosphocholine (POPC), modeling a mammalian lipid environment.^[^ ^72^ ^]^ Once the model membranes were built, six equilibrated peptide monomers, arranged in an antiparallel orientation, were placed 10 Å from the membrane surface and 10 Å apart from each other within each model membrane. The previously described two‐step protocol, along with the simulation setup, was applied. Free dynamics simulations under an ionic strength of 130 mm NaCl at 310 K in an isobaric–isothermal (NPT) ensemble were then performed for microsecond. The interactions of the peptides with the membrane surface and the degree of peptide immersion in the membrane were analyzed during the last 200 ns of the MD simulations. The interactions between peptides and the membrane during the final 200 ns of the MD simulations were analyzed based on their insertion profile and the number of peptide–membrane contacts. The insertion profile was determined by computing the density distribution of the z‐component of the peptide's COM relative to the average z‐coordinates of the phosphate groups in POPC, POPE/POPG, and POPG bilayers. Peptide–membrane contacts were quantified by counting instances where the distance between peptide and membrane atoms was ≤4.5 Å. All computations were performed using custom Python3 scripts based on MDAnalysis58, Matplotlib,^[^ ^73^ ^]^ and NumPy.^[^ ^74^ ^]^

## Conflict of Interest

The authors declare no conflict of interest.
